# Evaluation of coronary heights after Bio-Bentall using Piehler technique

**DOI:** 10.1093/icvts/ivaf150

**Published:** 2025-06-26

**Authors:** Kimihiro Kobayashi, Yoshinori Kuroda, Masahiro Mizumoto, Jun Hayashi, Shuto Hirooka, Kentaro Akabane, Tomonori Ochiai, Tetsuro Uchida

**Affiliations:** Second Department of Surgery, Faculty of Medicine, Yamagata University, 2-2-2 Iidanishi, Yamagata, 990-9585, Japan; Second Department of Surgery, Faculty of Medicine, Yamagata University, 2-2-2 Iidanishi, Yamagata, 990-9585, Japan; Second Department of Surgery, Faculty of Medicine, Yamagata University, 2-2-2 Iidanishi, Yamagata, 990-9585, Japan; Second Department of Surgery, Faculty of Medicine, Yamagata University, 2-2-2 Iidanishi, Yamagata, 990-9585, Japan; Second Department of Surgery, Faculty of Medicine, Yamagata University, 2-2-2 Iidanishi, Yamagata, 990-9585, Japan; Second Department of Surgery, Faculty of Medicine, Yamagata University, 2-2-2 Iidanishi, Yamagata, 990-9585, Japan; Second Department of Surgery, Faculty of Medicine, Yamagata University, 2-2-2 Iidanishi, Yamagata, 990-9585, Japan; Second Department of Surgery, Faculty of Medicine, Yamagata University, 2-2-2 Iidanishi, Yamagata, 990-9585, Japan

**Keywords:** aortic root geometry, Bio-Bentall, coronary height, valve-in-valve

## Abstract

The valve-in-valve procedure is an alternative to the redo surgery for structural valve deterioration following the Bio-Bentall. However, the risk of coronary obstruction and the feasibility of this approach remain unclear. Using computed tomography, we compared the aortic root geometry of 14 consecutive patients (13 true aortic aneurysms) with Bio-Bentall between April 2011 and April 2024 preoperatively and postoperatively. The Piehler technique was used in all coronary artery reconstructions. During the follow-up period, no reconstructive coronary events or valve-related reoperations were observed. The coronary height was 17.7 ± 5.6 mm preoperatively versus 18.8 ± 4.4 mm postoperatively for the left coronary artery (*P *= 0.49) and 15.4 ± 9.6 mm preoperatively versus 22.6 ± 7.4 mm postoperatively for the right coronary artery (*P *< 0.01). No patients at risk of coronary obstruction associated with valve-in-valve were identified. One case of severe aortic graft kinking due to excessive graft length, which could contraindicate the valve-in-valve procedure, was observed. Our findings suggested that the Piehler technique may facilitate future valve-in-valve implantation after Bio-Bentall because it allows for a higher-positioned orifice of the reconstructive coronary artery.

## INTRODUCTION

The valve-in-valve (ViV) procedure to treat structural valve deterioration (SVD) after surgical aortic valve replacement (SAVR) with bioprosthetic valves has become more widespread. ViV procedure is an alternative to re-SAVR, with lower early mortality rates, shorter hospital stays, and higher survival rates [1]. Although the use of bioprosthetic valves is increasing in Bentall procedures (Bio-Bentall) [2], similar to SAVR, the future SVD after the Bio-Bentall is an emerging concern. The ViV procedure is expected to be a valuable alternative to the technically challenging re-Bentall for SVD after Bio-Bentall; however, its feasibility has yet to be clarified. This study aimed to analyse changes in aortic root morphology, particularly coronary height, after the Bio-Bentall procedure using computed tomography (CT) and evaluate the risk of coronary obstruction in the ViV procedure.

## MATERIALS AND METHODS

### Ethical statement

This retrospective study was approved by the Institutional Review Board of Yamagata University Hospital (#2024–330, 9 March 2025), which waived the requirement for written informed consent from each patient.

### Patients and study design

Between November 2011 and March 2024, 14 of 15 consecutive Bio-Bentall cases at our hospital were retrospectively analysed, excluding one case without postoperative contrast-enhanced CT. Patient data were collected from the electronic medical records.

Our aortic root replacement procedures include the valve-sparing technique (David procedure) and the Bentall. In the Bentall, prosthetic valve selection criteria are based on the Japanese Circulation Society guidelines [3], with bioprosthetic valves recommended for patients aged 65 or older and mechanical valves for those under 60. Additionally, for coronary reconstruction, the Piehler technique [4] has been routinely employed for both left and right coronary arteries in all aortic root replacement since 2011.

### Bio-Bentall procedures (Video 1)

Under median sternotomy, standard cardiopulmonary bypass was established. The aortic root was dissected, the coronary buttons were mobilized, and the sinus of Valsalva (SoV) was excised, leaving an aortic remnant of approximately 8 mm. We dissected approximately 15 mm distal to the coronary artery orifice to prevent kinking of the coronary arteries. We employed a modified Bentall procedure using composite grafts with the flanged technique [5]. The composite graft was prepared by hand sewing a bioprosthetic valve into the aortic graft 5 mm larger than the valve. The surgeon decided to use a straight or Valsalva graft for the aortic graft. The inserted bioprosthesis was sutured 20 mm above the proximal end of the aortic graft with a running 5–0 polypropylene suture. A pledgeted 2–0 polyester braided sutures were placed at the aortic annulus from the outside in to fix the composite graft. To facilitate haemostasis, the aortic wall remnant was continuously sutured to the proximal end of the composite graft using a 5–0 polypropylene suture. Coronary arteries were reconstructed using the Piehler technique in all patients (Supplementary Fig. S1). An 8-mm Gelweave graft (Vascutek Ltd, Inchinnan, UK) was sutured to the left and right coronary artery buttons using continuous sutures with 5–0 polypropylene sutures. After trimming of Piehler grafts to a length of 5**–**10 mm, the grafts were anastomosed to a small side hole created in the aortic graft using continuous sutures with a 5–0 polypropylene suture in an end-to-side fashion. The new coronary orifice was placed slightly higher than the natural position to avoid kinking and twisting of the native coronary artery, targeting the position a few millimetres above the valve stent post. Especially because the right coronary artery was prone to kinking, new orifices were created higher than the left ones.

### CT analysis of aortic root

CT angiography using A 320-row multidetector CT scanner (Aquilion ONE, Toshiba Medical Systems, Tokyo, Japan) was performed pre- and 1 month postoperatively. CT acquisition was achieved using non-ECG-gated high-pitch CT of the aorta, which provides sufficient root analysis without artifacts caused by cardiac motion. Three researchers independently measured the aortic annular area, annular perimeter, SoV width, sinotubular junction (STJ) diameter, and coronary height. The risk of coronary obstruction associated with ViV procedure was evaluated using the VIVID classification [6]. Aortic graft morphology was assessed using postoperative CT, referring to previous reports [7], including the aorta-left ventricle (Ao-LV) angle, aortic graft angle, and aortic graft folding ratio. We defined an Ao-LV angle > 70°, aortic graft angle < 90°, and aortic graft folding ratio > 50% as an unsuitable aortic graft morphology for ViV procedure.

## STATISTICS

Categorical variables were expressed as numbers (%) and continuous variables as means ± standard deviation (SD) or median (interquartile range). Pre and postoperative measurements were compared using a paired t-test or Wilcoxon signed-rank test. Statistical significance was set at *P *< 0.05. All statistical analyses were performed with EZR (Saitama Medical Center, Jichi Medical University, Saitama, Japan), which is a graphical user interface for R (The R Foundation for Statistical Computing, Vienna, Austria) [8].

## RESULTS

**Figure 1:**
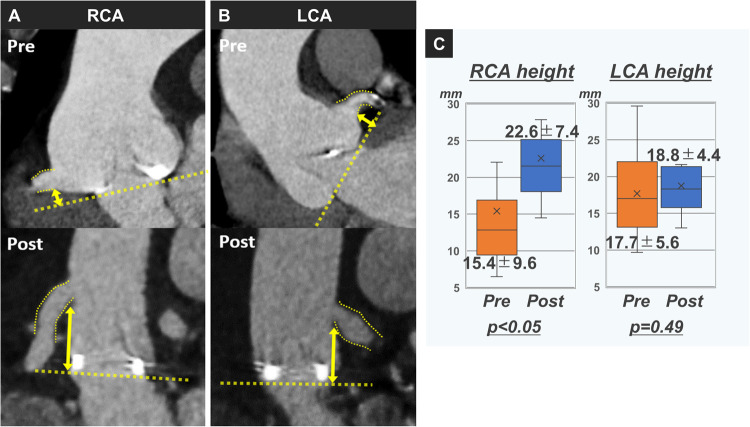
Computed tomographic data of preoperative and postoperative coronary height. **(A)** Reconstructed right coronary artery in a representative case of Bio-Bentall using the Piehler technique. **(B)** Reconstructed left coronary artery in a representative case of Bio-Bentall using the Piehler technique. **(C)** Comparison of the coronary heights before and after Bio-Bentall. LCA, left coronary artery; RCA, right coronary artery.

Preoperative and operative data are presented in Supplementary Table S1. The mean age of the 14 patients was 70.9 ± 6.1 years. In 13 patients (92.8%), a true aortic aneurysm was indicated for Bio-Bentall procedures. The clinical results are shown in Supplementary Table S2. During a follow-up period of a mean 36.3 ± 42.1 months (range: 1.0–149.0) with a follow-up rate of 100%, there were no SVDs, valve-related reoperations, or reconstructed coronary events. The bioprosthetic valves used in the composite grafts were Trifecta in one case, Avalus in one case, Carpentier-Edwards PERIMOUNT Magna in six cases, Inspiris in two cases, and Epic in four cases, and the size was 19 mm in one case, 21 mm in seven cases, 23 mm in five cases, and 25 mm in one case. CT data are presented in Table 1. The mean annular area after Bio-Bentall was 378.1 ± 56.1 mm2. The left coronary height was 17.7 ± 5.6 mm preoperatively versus 18.8 ± 4.4 mm postoperatively (*P *= 0.49). The right coronary height was 15.4 ± 9.6 mm preoperatively vs 22.6 ± 7.4 mm postoperatively (*P *< 0.01) (Fig. 1). All cases were classified as VIVID classification I, indicating that there were no cases at risk of coronary obstruction. The mean aortic graft angle was 112.0**°** ± 16.0**°**, the mean aortic graft folding ratio was 8.9 ± 14.7%, and the mean Ao-LV angle was 39.1**°** ± 11.9**°**. Severe aortic graft kinking was observed in one patient.

**Table 1:** Computed tomographic data of the aortic root geometry

Variables	Overall cohort (*n* = 14)		*P* value
	Preoperative	Postoperative	
Annular perimeter, mm	83.5 ± 13.2	63.9 ± 5.3	<0.001
Annular area, mm^2^	554.7 ± 161.0	378.1 ± 56.1	<0.001
SoV width, mm	44.2 ± 7.7	29.5 ± 1.7	<0.001
STJ diameter, mm	41.0 ± 9.1	28.8 ± 1.4	<0.001

Values are mean ± SD or *n* (%).

SoV, sinus of Valsalva; STJ, sinotubular junction.

## DISCUSSION

Studies examining aortic root morphology after the Bio-Bentall procedure are limited. In a study of 64 cases of the Bio-Bentall using the button technique, Werner *et al.* [2] reported that coronary height decreased significantly after surgery, with 59.4% of cases showing a coronary obstruction risk due to ViV procedures. Werner *et al.* argued that reducing the coronary height is inevitable for tension-free coronary reconstruction. However, in this study, the coronary height was sufficiently maintained using the Piehler technique, and no cases with a risk of coronary obstruction associated with ViV procedures were observed. Furthermore, while coronary reimplantation in reoperation is challenging due to severe adhesions, the Piehler technique may be especially effective in such situations [9], potentially achieving a higher coronary height. Our study included three redo cases. The drawbacks of the Piehler technique include an increased number of anastomotic sites and longer operative times. Nevertheless, the Piehler technique may be helpful for future ViV procedures because it can sufficiently secure coronary height compared to the button technique (Supplementary Fig. S2).

Additionally, severe aortic graft kinking due to excessive graft length was observed in one case of this study, which could be a contraindication for the ViV procedure. A study evaluating the aortic graft morphology after aortic root replacement reported severe aortic graft kinking in 8.6% of cases [7]. Cardiovascular surgeons should be mindful of aortic graft morphology because it could interfere with future ViV procedures.

## LIMITATIONS

This study has several limitations. First, it was a retrospective single-centre study. Second, the small number of enrolled patients is the most significant limitation. Therefore, the reliability and generalizability of the statistical analysis are limited. Third, several biases associated with the long study period can be considered. Finally, the anatomical changes after the Bio-Bentall may have been significantly influenced by our technical modifications, limiting the generalizability. Further studies involving more patients and multiple centres, and additional investigation of the effects of the Piehler technique on coronary perfusion and graft durability are considered necessary in the future.

## CONCLUSION

The Piehler technique may be optimal for coronary artery reconstruction in Bio-Bentall with future ViV optimization, allowing for a higher coronary height.

## Supplementary Material

ivaf150_Supplementary_Data

## Data Availability

The data underlying this article will be shared upon reasonable request to the corresponding authors.

## CLINICAL REGISTRATION

IRB No. 2024-330; Dated 9 March 2025 (Institutional Review Board of Yamagata University Hospital).
