# Supplementary material for: Evaluation of coronary heights after Bio-Bentall using Piehler technique
Source: Interdiscip Cardiovasc Thorac Surg. 2025 Jun 26;40(7):ivaf150. doi: 10.1093/icvts/ivaf150 (PMC12378618; doi:10.1093/icvts/ivaf150)
Supplement: ivaf150_Supplementary_Data [file ivaf150_Supplementary_Data.zip › Supplementary Table S2.docx]

Supplementary table S2. Postoperative outcomes

| Variable | Total (n=14) |
| --- | --- |
| In-hospital outcomes |  |
| 30-day mortality | 0 |
| In-hospital mortality | 2 (14.3) |
| Median ICU stay, days (range) | 5.0 (2.0-14.0) |
| Delirium | 3 (21.4) |
| Stroke | 0 |
| Reoperation for bleeding | 0 |
| Prolonged ventilation (> 72 hours) | 2 (14.3) |
| New need for dialysis | 1 (7.1) |
| Postoperative new atrial fibrillation | 4 (28.6) |
| Permanent pacemaker implantation | 0 |
| Deep sternal wound infection | 1 (7.1) |
| Late outcomes |  |
| Structural valve deterioration | 0 |
| Endocarditis | 0 |
| Myocardial infarction | 0 |
| Cardiac death | 0 |
| Non-cardiac death | 4 (28.6) |

Values are median (range) or n (%).

ICU: Intensive Care Unit.
