# Supplementary material for: Evaluation of coronary heights after Bio-Bentall using Piehler technique
Source: Interdiscip Cardiovasc Thorac Surg. 2025 Jun 26;40(7):ivaf150. doi: 10.1093/icvts/ivaf150 (PMC12378618; doi:10.1093/icvts/ivaf150)
Supplement: ivaf150_Supplementary_Data [file ivaf150_Supplementary_Data.zip › Supplementary Table S1.docx]

Supplementary table S1. Preoperative and operative data

| Variable | Total (n=14) |
| --- | --- |
| Age, years | 70.9 ± 6.1 |
| Female | 1 (7.1) |
| BMI, kg/m^2^ | 23.4 ± 4.9 |
| Indication of Bio-Bentall |  |
| Aneurysm | 13 (92.9) |
| Other | 1 (7.1) |
| Valve type |  |
| CEP Magna | 6 (42.9) |
| INSPIRIS | 2 (14.3) |
| Epic | 4 (28.6) |
| Trifecta | 1 (7.1) |
| Avalus | 1 (7.1) |
| Valve size, mm |  |
| 19 | 1 (7.1) |
| 21 | 7 (50.0) |
| 23 | 5 (35.8) |
| 25 | 1 (7.1) |
| Aortic graft size, mm |  |
| 24 | 1 (7.1) |
| 26 | 7 (50.0) |
| 28 | 5 (35.8) |
| 30 | 1 (7.1) |
| Valsalva graft | 5 (35.8) |

Values are mean ± SD or n (%).

BMI: body mass index; CEP: Carpentier-Edwards PERIMOUNT.
