# Supplementary figures and images for: Evaluation of coronary heights after Bio-Bentall using Piehler technique
Source: Interdiscip Cardiovasc Thorac Surg. 2025 Jun 26;40(7):ivaf150. doi: 10.1093/icvts/ivaf150 (PMC12378618; doi:10.1093/icvts/ivaf150)

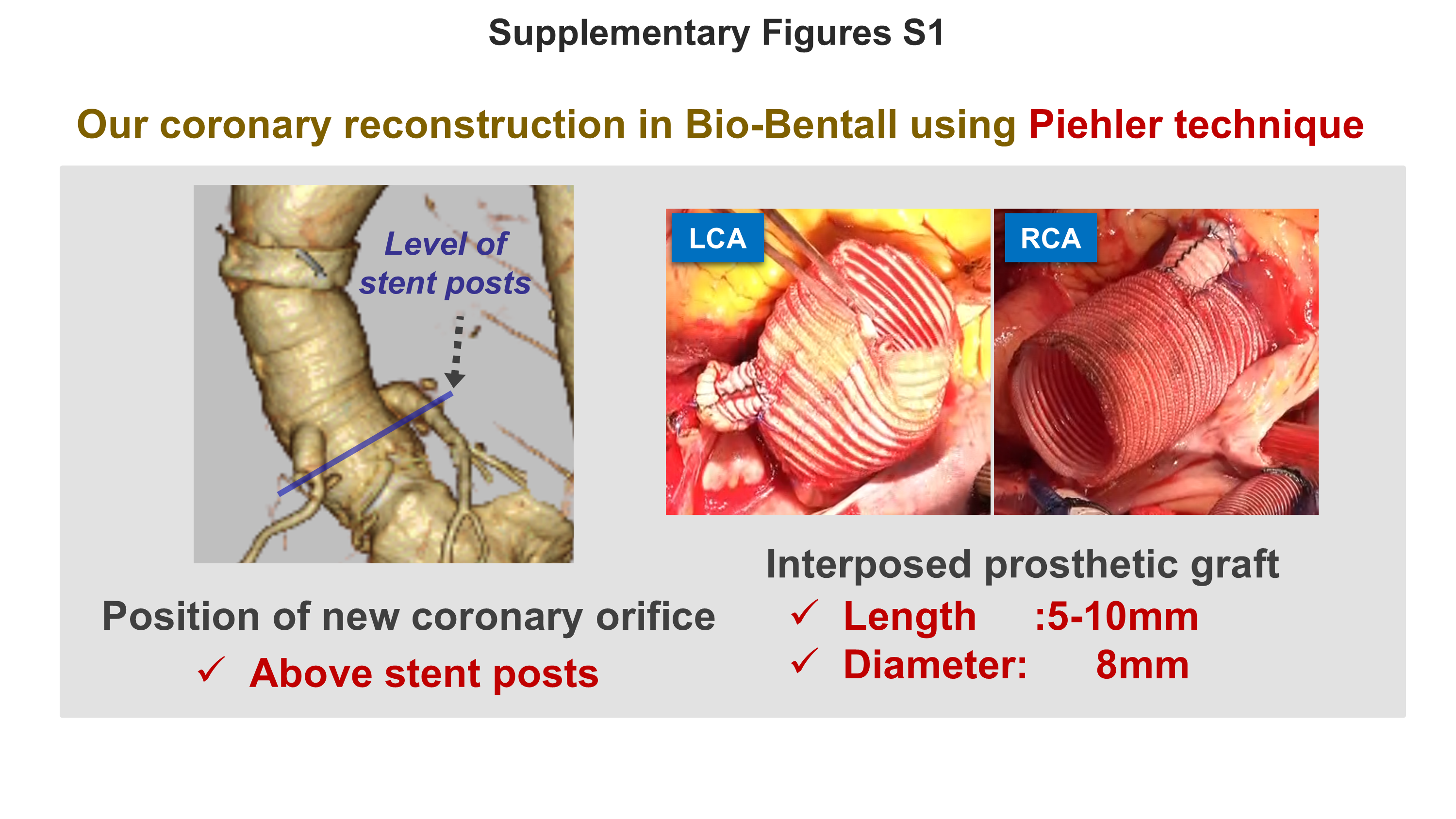

Supplement: ivaf150_Supplementary_Data [file ivaf150_Supplementary_Data.zip › Supplementary Figure S1.tif]

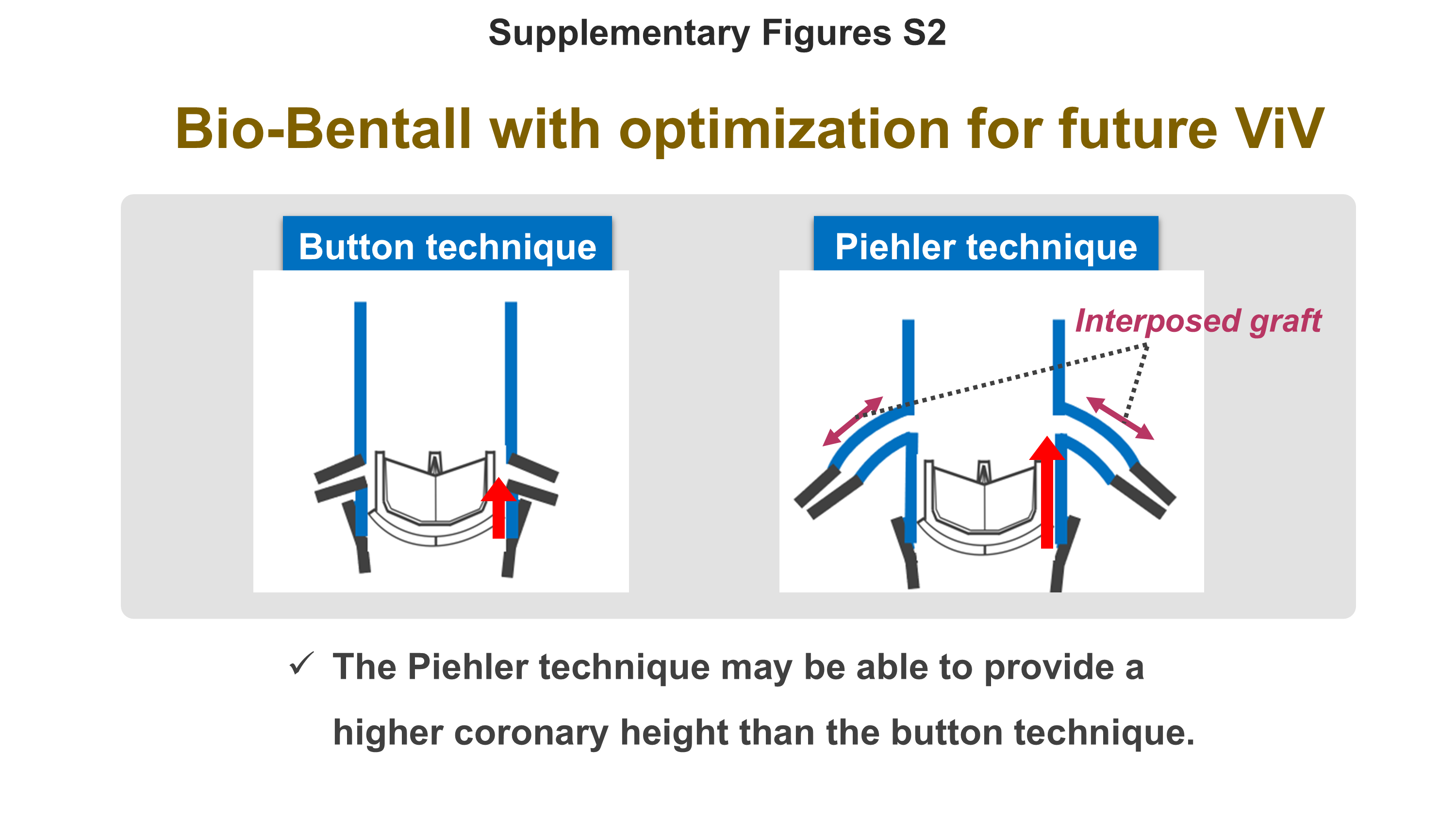

Supplement: ivaf150_Supplementary_Data [file ivaf150_Supplementary_Data.zip › Supplementary Figure S2.tif]
